# B4galnt2-mediated host glycosylation influences the susceptibility to *Citrobacter rodentium* infection

**DOI:** 10.3389/fmicb.2022.980495

**Published:** 2022-08-11

**Authors:** Abdulhadi Suwandi, Kris Gerard Alvarez, Alibek Galeev, Natalie Steck, Christian U. Riedel, José Luis Puente, John F. Baines, Guntram A. Grassl

**Affiliations:** ^1^Institute of Cell Biochemistry, Center of Biochemistry, Hannover Medical School, Hannover, Germany; ^2^Institute of Medical Microbiology and Hospital Epidemiology, Hannover Medical School and German Center for Infection Research (DZIF), Partner Site Hannover-Braunschweig, Hannover, Germany; ^3^Section of Evolutionary Medicine, Institute for Experimental Medicine, Kiel University, Kiel, Germany; ^4^Max Planck Institute for Evolutionary Biology, Plön, Germany; ^5^Institute of Microbiology and Biotechnology, University of Ulm, Ulm, Germany; ^6^Departamento de Microbiología Molecular, Instituto de Biotecnología, Universidad Nacional Autónoma de México, Cuernavaca, Morelos, Mexico

**Keywords:** B4galnt2, *Citrobacter*, intestinal inflammation, intestinal glycans, infectious colitis, gut colonization

## Abstract

Histo-blood group antigens in the intestinal mucosa play important roles in host–microbe interactions and modulate the susceptibility to enteric pathogens. The *B4galnt2* gene, expressed in the GI tract of most mammals, including humans, encodes a beta-1,4-N-acetylgalactosaminyltransferase enzyme which catalyzes the last step in the biosynthesis of the Sd(a) and Cad blood group antigens by adding an N-acetylgalactosamine (GalNAc) residue to the precursor molecules. In our study, we found that loss of *B4galnt2* expression is associated with increased susceptibility to *Citrobacter rodentium* infection, a murine model pathogen for human enteropathogenic *Escherichia coli.* We observed increased histopathological changes upon *C. rodentium* infection in mice lacking B4galnt2 compared to *B4galnt2*-expressing wild-type mice. In addition, wild-type mice cleared the *C. rodentium* infection faster than *B4galnt2^−/−^* knockout mice. It is known that *C. rodentium* uses its type 1 fimbriae adhesive subunit to bind specifically to D-mannose residues on mucosal cells. Flow cytometry analysis of intestinal epithelial cells showed the absence of GalNAc-modified glycans but an increase in mannosylated glycans in *B4galnt2-*deficient mice compared to *B4galnt2-*sufficient mice. Adhesion assays using intestinal epithelial organoid-derived monolayers revealed higher *C. rodentium* adherence to cells lacking *B4galnt2* expression compared to wild-type cells which in turn was reduced in the absence of type I fimbriae. In summary, we show that *B4galnt2* expression modulates the susceptibility to *C. rodentium* infection, which is partly mediated by fimbriae-mannose interaction.

## Introduction

*Citrobacter rodentium* is an extracellular enteric mouse pathogen causing transmissible murine colonic hyperplasia (Mundy et al., 2005). *C. rodentium* has been extensively used as a model for studying the human bacterial pathogens enteropathogenic *Escherichia coli* (EPEC) and enterohemorrhagic *E. coli* (EHEC; Croxen et al., 2013; Collins et al., 2014). EPEC is an important cause of diarrheal disease worldwide, whereas EHEC is a human pathogen responsible for the outbreaks of bloody diarrhea, hemorrhagic colitis and hemolytic uremic syndrome (Clarke et al., 2003; Nguyen and Sperandio, 2012). *C. rodentium*, EPEC and EHEC tightly adhere to the host intestinal epithelium and cause attaching and effacing (A/E) lesions, which are mediated by genes located in the locus of enterocyte effacement (LEE) pathogenicity island. The LEE encodes the outer membrane adhesin intimin, the translocated intimin receptor Tir, a type III secretion system (T3SS), effector proteins, chaperones and regulatory genes (Deng et al., 2004; Gaytán et al., 2016).

In the early stages of the infection process, these bacteria use various fimbriae/pili to adhere to and colonize the host intestinal epithelium (Caballero-Flores et al., 2015; Ribet and Cossart, 2015). Fimbriae are hair-like surface appendages that mediate diverse functions such as attachment to target cells, evasion of the host immune system, DNA transfer and twitching motility. Thus, fimbriae are important virulence factors (Proft and Baker, 2008). Type 1 fimbriae belong to the most studied fimbrial adhesins and are encoded by the *fim* gene cluster composed of a seven-cistron operon (*fimAICDFGH*). FimH, a lectin-like protein, is directly involved in binding to high-mannose oligosaccharides of eukaryotic cells (Jones et al., 1995; Foroogh et al., 2021).

The gastrointestinal tract’s epithelium and mucus layer are highly glycosylated (Corfield et al., 2001). These glycosylated layers are an important factor mediating host–microbe interaction. Intestinal commensal bacteria and pathogens can utilize glycosylated surfaces as molecular attachment sites or nutrient sources (Moran et al., 2011). The variation of epithelial glycan structures is often mediated by blood group-related glycosyltransferases and can influence susceptibility to infectious and chronic disease (Storry et al., 2011; Rausch et al., 2015; Suwandi et al., 2019; Galeev et al., 2021). Our previous study demonstrated that *Salmonella enterica* serovar Typhimurium can exploit host fucosylation in the intestine using its Std fimbriae *in vivo* and *in vitro* (Suwandi et al., 2019). In addition, glycosylation can also influence the microbiota, further affecting the protection against pathogens (Galeev et al., 2021). Furthermore, virulence gene expression by enteric pathogens can be regulated by glycans. For example, fucose, which is liberated by the commensal bacterium *Bacteroidetes thetaiotaomicron*, is sensed by EHEC and induces upregulation of virulence genes (Pacheco et al., 2012). The blood group glycosyltransferase β-1,4-N-acetylgalactosaminyltransferase 2 (B4galnt2) is responsible for the last step of the biosynthesis of the Sd(a)/Cad antigen by adding an N-acetylgalactosamine (GalNAc) residue *via* a β-1,4 linkage to a subterminal galactose residue substituted with an α-2,3-linked sialic acid (Galeev et al., 2021). We previously demonstrated that expression of *B4galnt2* in the gut resulted in differences in microbiota composition (Staubach et al., 2012), which contributes to susceptibility to *Salmonella* infection (Rausch et al., 2015).

In addition, while *C. rodentium* can bind to mannosylated proteins, there is no evidence for direct binding of *C. rodentium* to terminal GalNAc containing (B4galnt2-modified) glycans. Several other fimbriae including type IV pili (Bieber et al., 1998), Gcf fimbriae (Caballero-Flores et al., 2015) and Cfc (Mundy et al., 2003) were shown to be important for gut colonization. However, their molecular host targets are not well understood. To test whether B4galnt2-glycans affect the susceptibility to extracellular pathogens, we infected *B4galnt2* sufficient and deficient mice with *C. rodentium* and followed the infection kinetics. Here, we demonstrate that the absence of *B4galnt2* in the mouse intestine contributes to *C. rodentium* persistence and inflammation.

## Materials and methods

### Bacteria

For *in vivo* experiments, *C. rodentium* ICC169 was grown at 37°C with shaking in lysogeny broth (LB) overnight. For *in vitro* experiments, *C. rodentium* ICC169, *C. rodentium* DBS100, *C. rodentium* DBS100 *ΔfimA-H::Km* were grown in microaerobic stationary-phase cultures. Bacteria were grown for 8 h in LB with shaking at 37°C and then were diluted 1:100 into 10 ml fresh LB and incubated in 15-ml tubes in standing culture for 16 h at 37°C. LB supplemented with 100 μg/ml of kanamycin was used for *C. rodentium* DBS100 *ΔfimA-H::Km.*

### Construction of deletion mutants

The *C. rodentium* DBS100 *ΔfimA::Km, ΔfimH::Km* and *ΔfimA-H::Km* nonpolar deletion mutants were generated by the lambda-Red recombinase system (Datsenko and Wanner, 2000) using PCR fragments amplified with the corresponding specific primer pairs listed below and plasmid pKD4, which harbors a kanamycin resistance cassette, as the template. All mutations were confirmed by PCR amplification using primers flanking the deleted genes. Primers used in this study: **CrfimA-H1P1:** TGT TTA AAG GAA AAC AAT ATG AAA ATT AAG GCG TTG GCA ATT GTG TAG GCT GGA GCT GCT TC. **CrfimH-H2P2:** TTT CTC CGC CCG CCG GGA TTA CTG GTA AAC AAA GGT CAC CCC CAT ATG AAT ATC CTC CTT AG. **CrfimA-F:** TCT CCA CCT TTT TTC GCT TTC. **CrfimH-R:** AAA AAG ACG ATC AGC CGA CGA.

### Animal experiments

Mice heterozygous (*B4galnt2^+/−^*) and homozygous for B4galnt2 knock-out allele (*B4galnt2^−/−^*) were raised and housed together as littermates under SPF in the animal facility of the Leibniz Research Center Borstel, Germany. Standard chow (ssniff, Soest, Germany) and water were provided *ad libitum*. *B4galnt2^+/−^* and *B4galnt2^−/−^* littermates were orally gavaged with 2 × 10^9^ *C. rodentium* and were sacrificed by cervical dislocation at the indicated time points. Organs were homogenized in 1 ml PBS, serially diluted and plated on MacConkey agar to determine bacterial colonization.

### Ethical statement

Animal experiments were conducted upon approval from the Animal Care Committee of the Ministry of Energy, Agriculture, the Environment and Rural Areas of Schleswig-Holstein, Germany [approval number V244-7224.121.3 (99–10/10)].

### Histopathology

Organs were fixed in 10% neutral buffered formalin, dehydrated with ethanol, and embedded in paraffin. According to standard laboratory procedures, paraffin sections (5 μm) were deparaffinized, rehydrated and stained with hematoxylin and eosin (H&E). Scoring of histological changes was done in a blinded fashion by two independent pathologists. Pathological changes were assessed by evaluating the lumen, surface epithelium, mucosa and submucosa. (i) Lumen: necrotic epithelial cells (no cells: 0, scant: 1, moderate: 2) and the presence of neutrophils (no cells: 0, scant: 1, moderate: 2), (ii) Surface epithelium: desquamation (patchy: 1, diffuse: 2) and ulceration (absent: 0, present: 1), (iii) Mucosa: crypt abscesses [none: 0, rare (<15%) 1; moderate (15%–50%): 2]; inflammatory cell infiltrate (none: 0; scant: 1, moderate: 2), mononuclear cell infiltrate (one small aggregate: 0; more than one aggregate: 1; large aggregates: 2), and (iv) Submucosa: inflammatory cell infiltrate (none: 0, scant: 1, moderate: 2); mononuclear cell infiltrate (one small aggregate: 0, more than one aggregate: 1); edema, (none: 0, moderate: 1, severe: 2). Total pathology score is calculated as the sum of the four sub-scores.

### Immunofluorescence

Formalin-fixed paraffin-embedded tissue sections (5 μm) were deparaffinized, rehydrated and subjected to heat-induced antigen retrieval using 10 mM sodium citrate buffer (pH 6.0). About 2% normal goat serum (NGS) was added to block non-specific antibody binding. The following antibodies were used for immunostainings: anti-CD3 (Abcam), anti-F4/80 (Cell Signaling), anti-*E. coli* (Abcam) and fluorescently-labeled secondary antibodies (Invitrogen).

For quantification of CD3 and F4/80 positive cells, representative images of both stained sections were obtained using Zeiss Apotome 2 microscope (Zeiss). For CD3, the results are shown as median of positive CD3 stained cells per 10 crypts of each mouse. For F4/80, the results are shown as median of positive F4/80 stained cells per field of view (FOV) of each mouse. Quantification of total area covered by *C. rodentium* was determined in the cecal epithelial surface using ImageJ software version 1.52 using images taken at 200× magnification. The results are shown as median of area covered by *C. rodentium* per 10 crypts in mm^2^ of each mouse.

### Flow cytometry

Isolation of intestinal epithelial cells and lectin flow cytometry analysis from cecum and colon was performed as described in (Albers and Moore, 1996; Gracz et al., 2012) with modifications. To collect epithelial cells, colon luminal contents were flushed using cold PBS. Then, the colon was opened longitudinally, cut into 0.5 cm pieces and incubated in HBSS (without Ca^2+^ and Mg^2+^) with 5% FCS, 2 mM EDTA, 1 mM DTT and 10 mM HEPES at 37°C with shaking at 250 rpm for 15 min to release epithelium from the basement membrane. After removing the remnants of the intestinal tissue, the resulting cell suspension was centrifuged at 1,000× g for 5 min at 4°C. Cell pellets were washed and resuspended twice in DPBS with 10% FCS. Antibodies and lectins used for immunostaining and flow cytometry analysis are listed in Supplementary Table S1. Flow cytometry was performed using a MACSQuant Analyzer 10 (Miltenyi Biotec). Gating strategy is shown in Supplementary Figure S1. The data were analyzed using FlowJo v.10 software (TreeStar).

### Intestinal epithelial organoids and organoid-derived monolayer infection

Colon crypts were isolated from *B4galnt2^+/+^* and *B4galnt2^−/−^* mice and colonoids were cultivated as described (Miyoshi and Stappenbeck, 2013) with modifications. Briefly, mice were sacrificed by cervical dislocation. 1 cm of the proximal colon was removed, flushed with PBS, opened longitudinally and cut into small pieces. The resulting tissue fragments were washed with ice-cold PBS and incubated in ice-cold 10 mM EDTA in PBS for 90 min on an orbital shaker, after which tissue fragments were settled at the bottom of the tube. The supernatant was discarded and the tissue fragments (epithelial crypts) were resuspended in ice-cold PBS. Crypts were centrifuged for 5 min at 800 rpm at 4°C and pellets were resuspended in 1 ml ice-cold PBS. Around 100 crypts were resuspended in organoid medium [Advanced DMEM/F12 medium (Thermo Fischer Scientific) supplemented with 2 mM GlutaMax, 50% L-WRN-Supernatant (ATCC^®^ CRL3276™), 10 mM HEPES, 100 U/ml penicillin, 100 μg/ml streptomycin, B27 supplement, 50 ng/ml recombinant mouse epidermal growth factor (rm EGF), 500 nM A83-01 (Tocris), 10 μM SB202190 (Tocris), 10 nM Gastrin I (Tocris), 1 mM N-Acetyl-L-cysteine (Sigma), and 10 μM Y27623 (Tocris) and Matrigel (Corning)]. The crypt suspension was added into a well and incubated at 37°C with 5% CO_2_. After complete polymerization of the Matrigel, organoid medium was added to the well.

To perform an infection experiment, 2D monolayers were formed from 3D colonoids from *B4galnt2^+/+^* and *B4galnt2^−/−^* mice. 3D colonoids were resuspended in ice-cold PBS and centrifuged at 1,500 rpm for 10 min at 4°C. Pellets were resuspended in warm 0.05% trypsin/EDTA, incubated for 5 min at 37°C in a water bath and dissociated by pipetting. Crypt suspension was washed with ice-cold DMEM/10% FCS and resuspended in monolayer medium (Advanced DMEM/F-12, 50% L-WRN-Supernatant, 20% fetal bovine serum, 2 mM L-glutamine, 100 U/ml penicillin, 0.1 mg/ml streptomycin, 10 μM Y-27632, 50 ng/ml rm-EGF). Crypt suspension was seeded onto Transwell permeable supports (polyester; 6.5 mm diameter; 0.4 μm pore size; Corning) that had been coated with Matrigel diluted 1:40 in PBS for 2 h at 37°C. Monolayer medium was replaced every 2 days and monolayer barrier integrity was evaluated by measuring transepithelial electrical resistance (TEER) using a volt-ohmmeter (Millipore). On day 5 after seeding, the medium was changed to differentiation medium (Advanced DMEM/F-12, 5% L-WRN-Supernatant, 20% fetal bovine serum, 2 mM L-glutamine, 50 ng/ml rm-EGF, 5 μM DAPT). The differentiation medium was changed daily for the next 2 days, and TEER was measured. 2D monolayers were infected with either *C. rodentium* ICC169, *C. rodentium* DBS100 or *C. rodentium* DBS100 *ΔfimA-H::Km* (10^8^ bacteria per Transwell), incubated for 30 min at 37°C, washed four times with PBS, and lysed in PBS containing 1% (v/v) Triton X-100. The number of adherent bacteria was determined by plating serial dilutions on LB plates.

### Statistical analyses

All data were analyzed using GraphPad Prism V6.0d software. Statistical analyses were performed using one-way analysis of variance followed by Tukey’s multiple comparison test or unpaired t test as indicated. Graphs display the mean values ± SEM, unless stated otherwise.

## Results

### B4galnt2 expression influences susceptibility to *Citrobacter rodentium* infection in mice

**Figure 1:**
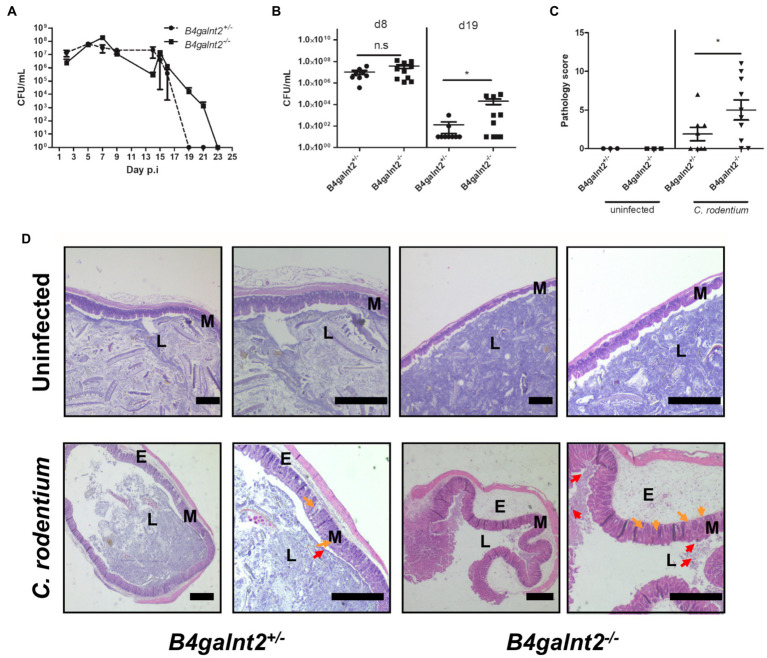
*B4galnt2* expression affects mouse susceptibility to *Citrobacter rodentium*. *B4galnt2*^*+*/−^ and *B4galnt2*^*−*/−^ mice were orally infected with *C. rodentium* and intestinal colonization was analyzed by plating homogenates of fecal pellets on MacConkey agar. Pooled data of two independent experiments (total number of mice per group = 9–10). **(A)** *B4galnt2*^*+*/−^ and *B4galnt2*^*−*/−^ mice were orally infected with *C. rodentium* and groups of mice were killed at day 8 and day 19 p.i. *C. rodentium* loads were determined in cecum by plating homogenates on MacConkey agar. **(B)** Histology scoring in cecum sections from uninfected and *C. rodentium* infected *B4galnt2*^+/−^ and *B4galnt2*^−/−^ mice at day 8 p.i. **(C)** H&E staining of cecum tissue sections of uninfected mice and of *C. rodentium* infected mice at day 8 p.i. **(D)** Uninfected *B4galnt2^+/−^* and *B4galnt2^−/−^* mice had normal tissue architecture and no signs of pathological changes. Detached epithelial cells (red arrows) in the cecal lumen (L), inflammatory cells (orange arrows) in the mucosa (M) and submucosal edema (E). Original magnifications: 20× and 40×. Scale bars = 500 μm. Graphs are pooled data of two independent infection experiments. Each dot corresponds to an individual animal. Mean ± SEM is shown. Unpaired *t*-test. ^*^p < 0.05.

**Figure 2:**
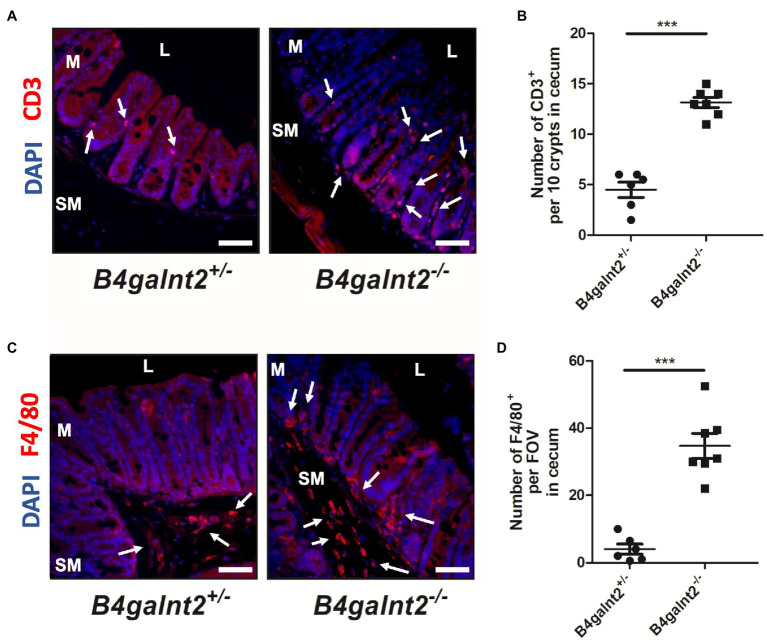
Increased infiltration of immune cells in B4galnt2-deficient mice after *Citrobacter rodentium* infection. *B4galnt2*^*+*/−^ and *B4galnt2*^*−*/−^ mice were orally infected with *C. rodentium*. Cecum sections at day 8 p.i. were stained with antibodies to CD3 (red; **A,B**) and F4/80 (red; **C,D**). Nuclei were stained with DAPI (blue). CD3 and F4/80 positive cells are indicated by white arrows. L, cecal lumen; M, mucosa; SM, submucosa. Quantification of CD3 positive cells per 10 crypts **(B)** and F4/80 positive cells per field of view (FOV; **D**). Original magnification: 200 ×. Scale bars = 50 μm. Mean ± SEM is shown. Unpaired *t*-test. ^***^p < 0.001.

*B4galnt2* encodes a blood group-related glycosyltransferase and wild mice show signs of balancing selection at this locus, which may be associated with trade-offs between allelic variants at this locus (Linnenbrink et al., 2011). *B4galnt2* is expressed by intestinal epithelial cells and we previously demonstrated that *B4galnt2*-deficient mice are more resistant to infection with the intestinal facultative intracellular pathogen *S.* Typhimurium (Rausch et al., 2015). In order to test if *B4galnt2*-dependent glycans affect the susceptibility to other intestinal pathogens, we orally infected *B4galnt2^+/−^* and *B4galnt2^−/−^* mice with *C. rodentium* and followed the colonization kinetics by plating homogenates of fecal pellets. We observed similar colonization of *C. rodentium* in *B4galnt2^−/−^* and *B4galnt2^+/−^* mice during early time points after infection. However, on day 19 post infection (p.i.) *B4galnt2^+/−^* mice cleared the infection whereas clearance of bacteria in *B4galnt2^−/−^* mice was delayed until day 23 p.i. (Figure 1A). In order to evaluate bacterial colonization in the intestine, mice were sacrificed on days 8 and 19 p.i. and bacterial burden was quantified in homogenized ceca. While bacterial loads were similar in *B4galnt2^+/−^* and *B4galnt2^−/−^* mice at 8 days p.i., higher bacterial loads were observed in *B4galnt2^−/−^* mice compared to *B4galnt2^+/−^* mice at 19 days p.i. (Figure 1B). Next, we analyzed histopathological changes in the intestines of infected mice. We found that ceca of *B4galnt2^−/−^* mice were more inflamed and had higher numbers of detached epithelial cells in the cecal lumen, increased inflammatory cell infiltration within the intestinal mucosa, as well as larger submucosal edema in comparison to *B4galnt2^+/−^* mice at 8 days p.i. (Figures 1C,D). Furthermore, cecum tissue sections were analyzed for the presence of CD3 positive cells (marker for T cells) and F4/80 positive cells (macrophages) by immunohistochemical staining. Significantly higher numbers of CD3 positive cells were detected in intestinal mucosa and submucosa of *B4galnt2^−/−^* mice compared to *B4galnt2^+/−^* mice (Figures 2A,B). Similarly, higher numbers of F4/80 positive cells were detected in the submucosa of *B4galnt2^−/−^* mice compared to *B4galnt2^+/−^* mice (Figures 2C,D). Thus, these data demonstrate that mice lacking B4galnt2 glycans are less proficient in clearing *C. rodentium* and suffer from increased intestinal inflammation.

### *B4galnt2* expression affects overall glycoprotein composition of the intestinal epithelium

**Figure 3:**
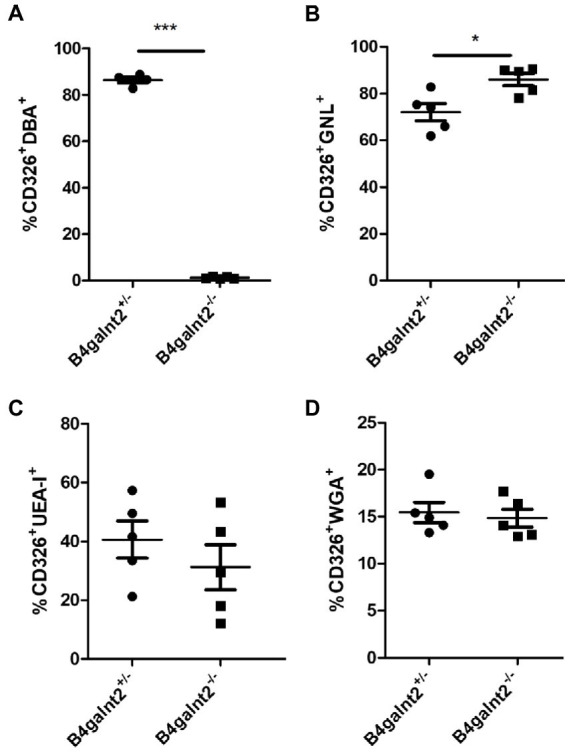
Flow cytometry analysis of lectin-stained intestinal epithelial cells. Intestinal epithelial cells from *B4galnt2*^*+*/−^ and *B4galnt2*^*−*/−^ mice were isolated, stained with different lectins and analyzed by flow cytometry. **(A)** Percentage of DBA^+^ (CD326^+^CD45^−^DBA^+^), **(B)** GNL^+^(CD326^+^CD45^−^GNL^+^), **(C)** UEA-I^+^ (CD326^+^CD45^−^UEA-I^+^), and **(D)** WGA+ (CD326^+^CD45^−^WGA^+^) intestinal epithelial cells (*n* = 4–5). Mean ± SEM is shown. Unpaired *t*-test. ^*^p < 0.05; ^***^p < 0.001.

*C. rodentium* adheres to the heavily glycosylated intestinal epithelium. To investigate whether *B4galnt2* expression influences the glycoprotein composition in the mucosal surface, which may alter susceptibility to *C. rodentium* infection, flow cytometry analysis of CD326-positive intestinal epithelial cells stained with different lectins was performed. As expected, most intestinal epithelial cells of *B4galnt2^+/−^* mice stained positive with *Dolichos biflorus* agglutinin (DBA), a lectin that specifically binds β-1,4 linked GalNAc residues. This DBA signal was absent in *B4galnt2^−/−^* mice (Figure 3A). In addition, we found that a higher percentage of epithelial cells from *B4galnt2^−/−^* mice stained positive with *Galanthus nivalis* lectin (GNL), binding terminal mannose, in comparison to epithelial cells from *B4galnt2^+/−^* mice (Figure 3B). Furthermore, no statistically significant differences were found when stained with *Ulex europaeus* agglutinin I (UEA-I) or wheat germ agglutinin (WGA), which bind fucosylated residues or N-acetylglucosamine-modified glycans, respectively (Figures 3C,D).

### B4galnt2-glycans influence *Citrobacter rodentium* adhesion in organoid-derived primary epithelial monolayer

**Figure 4:**
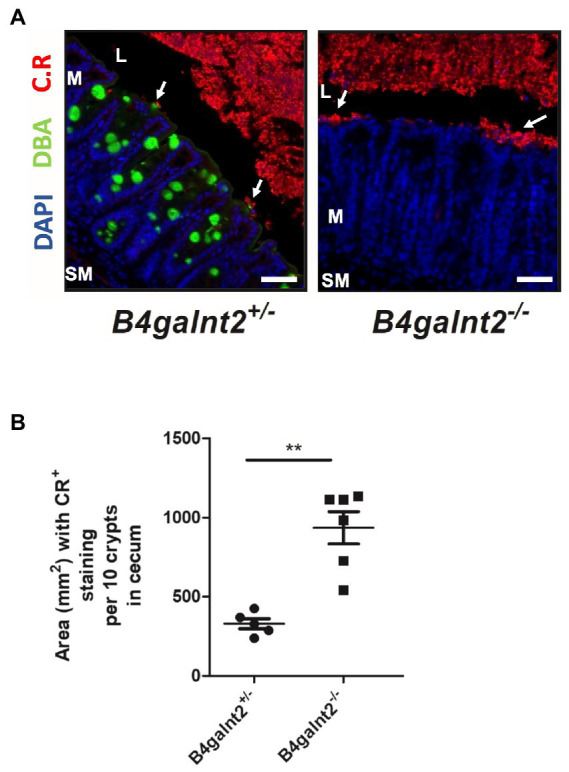
B4galnt2 affects adhesion of *Citrobacter rodentium* to the epithelium. **(A)** Immunofluorescence staining and **(B)** quantification of total area of epithelium covered by *C. rodentium* (white arrows) at day 8 p.i.. Nuclei were stained with DAPI (blue), B4galnt2 glycans were visualized by using fluorescein-labeled DBA (green), and *C. rodentium* (C.R) staining is shown in red. Original magnification: 200×. Scale bars = 50 μm. L, cecal lumen; M, mucosa; SM, submucosa. Mean ± SEM is shown. Mann–Whitney test. ^**^p < 0.01.

**Figure 5:**
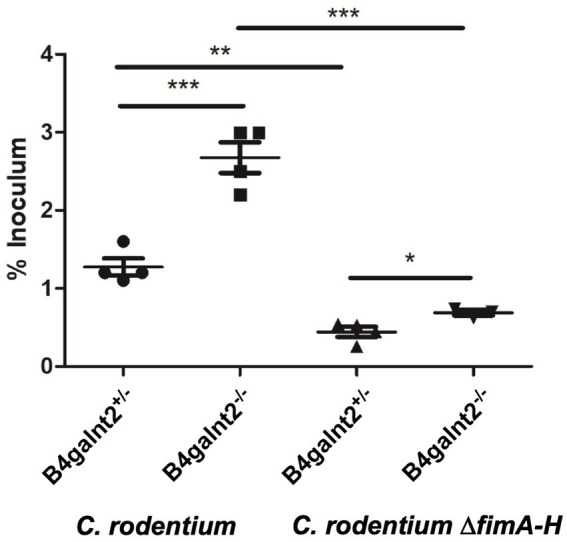
*B4galnt2* expression affects bacterial adhesion to epithelial cells which is in part dependent on type 1 fimbriae. Organoids from *B4galnt2*^+/+^ and *B4galnt2*^−/−^ mice were dissociated, seeded on Transwell filter inserts and grown into a differentiated epithelial monolayer. Organoid-derived epithelial monolayers were infected with WT *Citrobacter rodentium* and *C. rodentium* Δ*fimA-H* deletion mutant for 30 min. Unbound bacteria were removed by washing with PBS, and bound bacteria were enumerated after lysis of the cells by plate counting on LB agar. Graphs display a representative out of two independent experiments. Mean ± SEM is shown. One-way ANOVA with Tukey’s multiple comparison test. ^*^p < 0.05, ^**^p < 0.01, ^***^p < 0.001.

To assess the spatial distribution of *C. rodentium* on the intestinal epithelium, we visualized the bacteria by immunofluorescence staining (bacteria are shown in red). B4galnt2-glycans were stained with DBA lectin (green). Note that while DBA staining is very strong in goblet cells, there is also positive DBA staining in the apical membrane of most epithelial cells. The cecum epithelial surface of *B4galnt2^−/−^* mice was in large parts covered by *C. rodentium*, while there were bigger gaps on the epithelium of *B4galnt2^+/−^* mice (Figures 4A,B). To further investigate whether B4galnt2 glycans influence the interaction of *C. rodentium* with the intestinal epithelium, we cultivated mouse-derived epithelial organoids *in vitro*. Primary colon epithelial cells from *B4galnt2^+/+^* and *B4galnt2^−/−^* mice were isolated, cultivated and expanded as organoids embedded in matrigel. These colonoids were dissociated and seeded onto Transwell filters to form a monolayer. After 8 days of cultivation, polarized monolayers were infected with *C. rodentium* and adherent bacteria were quantified after 30 min. Significantly higher bacterial loads were found adherent to the *B4galnt2^−/−^* monolayer compared to *B4galnt2^+/+^* cells (Figure 5). This data shows that the absence of epithelial *B4galnt2* expression facilitates adhesion of *C. rodentium*.

### *Citrobacter rodentium* type 1 fimbria plays a role in adhesion to *B4galnt2^−/−^* epithelial cells

*Citrobacter rodentium* employs fimbriae to adhere and colonize the intestinal epithelium. The *fim* operon encodes type 1 fimbriae (T1F) and the adhesin FimH, a lectin-like protein located at the tip of the fimbrial structure, has been shown to bind to terminal mannose residues on epithelial glycoproteins (Jones et al., 1995; Schembri et al., 2001). To analyze the role of T1F in the interaction of *C. rodentium* with the intestinal epithelium, we infected *B4galnt2^+/+^* and *B4galnt2^−/−^* organoid-derived epithelial monolayers with WT *C. rodentium* and its isogenic mutant lacking the *fim* operon (*C. rodentium ΔfimA-H*) for 30 min. After washing with PBS, bacterial loads were quantified. Significantly more *C. rodentium* bacteria adhered to the epithelial monolayers from *B4galnt2^−/−^* mice compared to the epithelium from *B4galnt2^+/+^* mice. After infection with *C. rodentium ΔfimA-H*, fewer bacteria bound to both *B4galnt2^−/−^* and *B4galnt2^+/+^*epithelial monolayers (Figure 5). In addition, the difference in binding to *B4galnt2^−/−^* and *B4galnt2^+/+^*epithelial cells became smaller, indicating a role of T1F in binding to B4galnt2-dependent glycans. Taken together, our *in vivo* data demonstrate that the lack of B4galnt2 in the intestine delayed the clearance of *C. rodentium* and increased intestinal inflammation. Furthermore, our *in vitro* data suggests that T1F-mannose interaction contributes to adhesion to the intestinal epithelium.

## Discussion

Glycosylation is a post-translational modification of proteins and lipids and is involved in regulating a wide range of cellular and molecular processes from bacteria to humans. This enzyme-directed process has crucial roles in (patho)physiological processes, for example, in infectious diseases, inflammatory diseases, cancer and aging (Larsson et al., 2011; Moran et al., 2011; Dall’Olio et al., 2013; Pinho and Reis, 2015). The gastrointestinal tract of humans and other mammals is covered by a glycosylated mucus layer that protects gut epithelial cells from chemical, biological, and physical insults and is continuously renewed. Glycan-lectin interaction is one of the key events in initiating an infection process.

Here, we demonstrate that B4galnt2 deficient mice that lack terminal GalNAc but possess more mannosylated glycoproteins are characterized by increased susceptibility to *C. rodentium* infection, develop stronger intestinal inflammation and show delayed bacterial clearance compared to B4galnt2 sufficient mice. *In vitro*, more *C. rodentium* bacteria adhere to *B4galnt2^−/−^* epithelial monolayers than *B4galnt2^+/+^* epithelial monolayers. These data suggest that this phenotype results from a direct interaction between *C. rodentium* and host cell structures that are more abundantly expressed or exposed in cells deficient in B4galnt2.

*Citrobacter rodentium* must adhere tightly to the host epithelium in order to translocate its effector proteins into the host cells. However, the early steps leading to adherence of *C. rodentium* to the intestinal epithelium and to establishing infection *in vivo* are poorly understood. For example, it was previously shown that overexpression of the glycoprotein C type lectin 2 (Clec2) increases *C. rodentium* adherence to epithelial cells (Woo et al., 2019). The authors also demonstrated that Clec2 expression is dependent on the intestinal microbiota, thereby modifying the susceptibility to infection with *C. rodentium*. However, it is currently unknown whether Clec2 can be modified by B4galnt2.

*Citrobacter rodentium* infection results in complex changes in the glycosylation pattern of the colon epithelium (Ahmed et al., 2018). Together with intestinal inflammation, these changes in available glycoproteins are responsible for the development of dysbiosis, and thus epithelial binding sites for *C. rodentium* might increase or decrease.

We have previously demonstrated that B4galnt2-dependent glycans only mildly affect the adhesion of the facultative intracellular pathogen *S.* Typhimurium to epithelial cells. However, fecal microbiota transplantation experiments demonstrated that B4galnt2 modulates the intestinal microbiota composition, conferring susceptibility to intestinal inflammation, while B4galnt2-deficient mice were more resistant to *S.* Typhimurium infection, thus suggesting that epithelial B4galnt2 expression facilitated epithelial invasion of *S*. Typhimurium (Rausch et al., 2015).

B4galnt2 modifies the Tamm-Horsfall glycoprotein (THGP). THGP is highly sialylated and mannosylated and thus contains binding sites for S type and type-1 fimbriated uropathogenic *E. coli*, which recognize sialyl and mannose residues, respectively. The addition of GalNac residues by B4galnt2 thus masks its binding sites for *E. coli* (Serafini-Cessi et al., 2005). Similarly, several studies showed that B4galnt2 can modify the sialylated surface receptors for avian influenza strains, thereby masking the attachment site for these viruses and inhibiting infection (Heaton et al., 2017; Wong et al., 2019), further emphasizing an important function of B4galnt2-glycosylation for infection with bacterial and viral pathogens.

Our adhesion experiments using organoid-derived monolayers obtained from *B4galnt2^−/−^* and *B4galnt2^+/+^* mice showed that more *C. rodentium* bacteria adhere to the *B4galnt2^−/−^* epithelium. In addition, lectin flow cytometry of intestinal epithelial cells showed a higher percentage of GNL positive cells (specifically binding to terminal mannose residues) in B4galnt2-deficient mice compared to B4galnt2-sufficient mice. Different species of the family *Enterobacteriaceae*, including *Citrobacter* spp., *Escherichia* spp. and *S. enterica* have been shown to possess type 1 fimbriae, one of the most common adhesins, to initialize attachment to the host intestinal mucosa (Sarowska et al., 2019). These thin filamentous structures are responsible for recognizing and binding high-mannose oligosaccharides in glycoconjugates at the host cell surface. The proteins responsible for T1F biogenesis are encoded by the polycistronic *fim* operon encoding the structural components (FimA, FimF, FimG and FimH) and a chaperone-usher assembly system (FimC and FimD). Several studies suggested that the FimH subunit is responsible for mediating mannose-specific adherence (Krogfelt et al., 1990; Jones et al., 1995). There is also some evidence for the direct binding of *E. coli* F1C fimbriae to GalNAc-containing glycans (Day et al., 2021). However, it remains to be seen if these glycans are B4galnt2-modified.

Several other fimbriae, including type IV pili (Bieber et al., 1998), Gcf fimbriae (Caballero-Flores et al., 2015) and Cfc (Mundy et al., 2003) were shown to be important for gut colonization; however, their molecular host targets are not well understood. The difference in binding to organoid-derived monolayers from *B4galnt2^+/+^* and *B4galnt2^−/−^* mice was much smaller when the cells were infected with the *C. rodentium* ∆*fimA-H* mutant compared to infection with the wild-type bacteria; therefore, we conclude that fimbriae-mannose binding plays an important role for *C. rodentium* binding to the *B4galnt2^−/−^* epithelium.

The *C. rodentium* genome encodes 19 fimbrial operons, of which 14 belong to the chaperone-usher family (Petty et al., 2010). Most of these operons are not expressed under *in vitro* growth conditions or the feces from infected mice (Caballero-Flores et al., 2015; Smith et al., 2016); however, as mentioned above, there is evidence that some play a role during the colonization process, suggesting that specific *in vivo* signals allow their differential expression. In the context of the results presented here, it is tempting to speculate that the multifactorial events that shape microbiota composition, such as B4galnt2 expression, may also establish variable scenarios where a different display of fimbriae is expressed by *C. rodentium* during the infection, therefore, determining different colonization levels.

The intestinal microbiota plays an important role in *C. rodentium* infection. For example, commensal bacteria that induce a Th17 response with increased levels of IL-17 and IL-22 production results in resistance to *C. rodentium* infection (Ivanov et al., 2009; Willing et al., 2011; Collins et al., 2014). In addition, microbiota also controls mucin production and the thickness of the inner mucus layer, thereby restricting access of *C. rodentium* to the epithelium (Bergstrom et al., 2010; Wlodarska et al., 2011). In this regard, we have not observed any influence of B4galnt2 on mucus thickness (Rausch et al., 2015). Commensal bacteria can also directly affect the binding of *C. rodentium* to the epithelium by downregulating host cell receptors such as Clec2e (Woo et al., 2019); however, as mentioned above, we do not know if B4galnt2 can glycosylate Clec2e nor if this would affect *C. rodentium* binding to this receptor. It remains to be seen if the microbiota composition, the diet, or the genetic background, among other variables, may mediate different levels of B4galnt2 expression in intestinal epithelial cells and thus a variable role of T1F in cell adherence and colonization.

In summary, we demonstrate that in the absence of the *B4galnt2* gene, a higher exposure of terminal mannose residues on the intestinal epithelium is observed, which correlates with increased adherence of *C. rodentium via* type 1 fimbriae. Therefore, we conclude that *C. rodentium* infection of *B4galnt2^−/−^* mice results in increased pathological changes and prolonged colonization due to a type 1 fimbriae-mannose dependent mechanism.

## Data availability statement

The original contributions presented in the study are included in the article/Supplementary material, further inquiries can be directed to the corresponding author.

## Ethics statement

Animal experiments were conducted upon approval from the Animal Care Committee of the Ministry of Energy, Agriculture, the Environment and Rural Areas of Schleswig-Holstein, Germany [approval number V244-7224.121.3 (99-10/10)].

## Author contributions

AS, NS, JP, JB, and GG conceived and designed the experiments. AS, KA, AG, and NS performed the experiments. CR and JP contributed reagents, materials, and tools. AS, KA, AG, NS, and GG analyzed the data. AS, KA, AG, NS, CR, JP, JB, and GG wrote and edited the paper. All authors contributed to the article and approved the submitted version.

## Funding

This work was funded by the DFG priority program SPP1656/1 and SPP1656/2 to GG and JB, the DFG Cluster of Excellence 2167 “Precision Medicine in Chronic Inflammation (PMI)” (grant no. EXC2167), and by DGAPA-PAPIIT IN213516 and CONACyT FC-2015-2/950 to JP. AG and KA were supported by the Center for Infection Biology (ZIB) at the Hannover Biomedical Research School (HBRS). KA was supported by the Graduate School Scholarship Program (GSSP) from the German Academic Exchange Service (DAAD).

## Conflict of interest

The authors declare that the research was conducted in the absence of any commercial or financial relationships that could be construed as a potential conflict of interest.

## Publisher’s note

All claims expressed in this article are solely those of the authors and do not necessarily represent those of their affiliated organizations, or those of the publisher, the editors and the reviewers. Any product that may be evaluated in this article, or claim that may be made by its manufacturer, is not guaranteed or endorsed by the publisher.
