# Supplementary material for: B4galnt2-mediated host glycosylation influences the susceptibility to Citrobacter rodentium infection
Source: Front Microbiol. 2022 Aug 11;13:980495. doi: 10.3389/fmicb.2022.980495 (PMC9403859; doi:10.3389/fmicb.2022.980495)
Supplement: Supplementary file 1 [file Image_1.pdf]

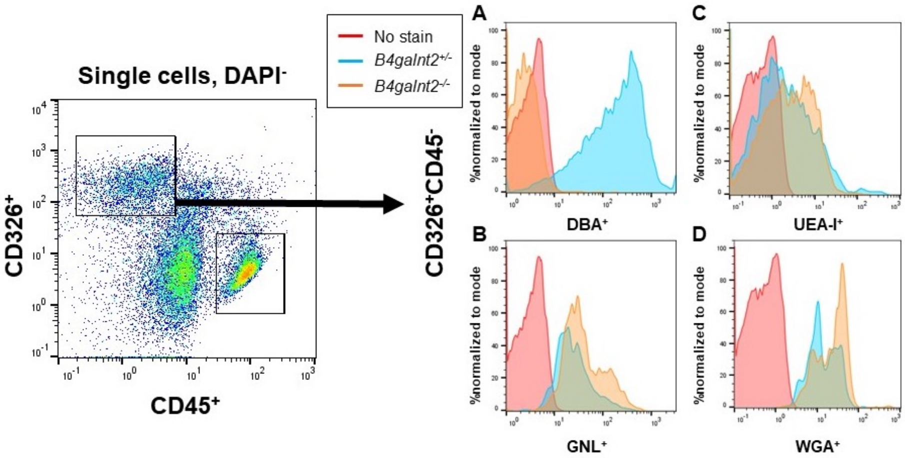

Figure S1. Flow cytometry analysis of lectin-stained intestinal epithelial cells. Intestinal epithelial cells from *B4galnt2*$^{+/-}$ and *B4galnt2*$^{-/-}$ mice were isolated, stained with different lectins as indicated and analyzed by flow cytometry. Flow cytometry gating strategy for intestinal epithelial cells. Single cells (FSC-A vs FSC-H), live cells (DAPI^{-}), CD326^{+}CD45^{-} population were gated. (A) Percentage of DBA^{+} (CD326^{+}CD45^{-}DBA^{+}), (B) GNL^{+}(CD326^{+}CD45^{-}GNL^{+}), (C) UEA-I^{+} (CD326^{+}CD45^{-}UEA-I^{+}) and (D) WGA+ (CD326^{+}CD45^{-}WGA^{+}) in intestinal epithelial cells (n = 4–5).
