# Supplementary material for: B4galnt2-mediated host glycosylation influences the susceptibility to Citrobacter rodentium infection
Source: Front Microbiol. 2022 Aug 11;13:980495. doi: 10.3389/fmicb.2022.980495 (PMC9403859; doi:10.3389/fmicb.2022.980495)
Supplement: Supplementary file 2 [file Table_1.pdf]

Table S1: Antibodies and Lectins used in this study

Antibodies/lectins	Source	Cat. Nr	RRID
Anti-CD3 antibody	Abcam	ab5690	AB_305055
Anti-myeloperoxidase (MPO)	Thermo Fischer Scientific	RB-373-A	AB_59597
Anti-F4/80	Cell Signaling	70076	AB_2799771
Anti-E. coli LPS antibody [2D7/1]	Abcam	ab35654	AB_732222
Anti-CD326 (EpCAM)[G8.8], APC	eBioscience	17-5791-82	AB_2716944
Anti-CD45, APC/Cyanine7	BioLegend	103116	AB_312981
Dolichos Biflorus Agglutinin (DBA), Fluorescein	Vector Laboratories	FL-1031	AB_2336394
Ulex Europaeus Agglutinin I (UEA I), Rhodamine	Vector Laboratories	RL-1062-2	AB_2336769
Galanthus Nivalis Lectin (GNL), Fluorescein	Vector Laboratories	FL-1241	AB_2336470
Wheat Germ Agglutinin (WGA), Rhodamine	Vector Laboratories	RL-1022	AB_2336871
